# Inferior vena cava diameters and collapsibility index reveal early volume depletion in a blood donor model

**DOI:** 10.1186/s13089-015-0034-4

**Published:** 2015-11-04

**Authors:** Paolo Pasquero, Stefano Albani, Elena Sitia, Anna Viola Taulaigo, Lorenzo Borio, Paola Berchialla, Franco Castagno, Massimo Porta

**Affiliations:** Department of Medical Sciences, University of Turin, Corso AM Dogliotti 14, 10126 Turin, Italy; Department of Clinical and Biological Sciences, University of Turin, Turin, Italy; Blood Bank, AOU Città della Salute e della Scienza, Turin, Italy

**Keywords:** Ultrasound, Blood loss, Inferior vena cava, Collapsibility index, Volume depletion

## Abstract

**Background:**

Changes of volume status can be readily inferred from variations in diameter of the inferior vena cava (IVC) measured by ultrasound. However the effect of IVC changes following acute blood loss are not fully established. In this study, three different approaches to measuring IVC variables were compared in healthy blood donors, as a model of acute volume depletion, in order to establish their relative ability to detect acute blood loss.

**Methods:**

Inspiratory and expiratory IVC diameters were measured before and after blood donation in hepatic long axis, hepatic short axis and renal short axis views using a 2–5 MHz curvilinear probe. All measurements were recorded and examined in real-time and post-processing sessions.

**Results:**

All windows performed satisfactorily but the renal window approach was feasible in only 30 out of 47 subjects. After blood donation, IVC diameters decreased in hepatic long axis, hepatic short axis and renal short axis (expiratory: −19.9, −18.0, −26.5 %; CI 95 %: 14.5–24.1; 13.1–22.9; 16.0–35.9, respectively) (inspiratory: −31.1, −31.6, −36.5 %; CI 95 %: 21.3–40.1; 18.8–45.2; 23.4–46.0, respectively), whereas the IVC collapsibility index increased by 21.6, 22.6 and 19.3 % (CI 95 %: 11.6–42.9; 18.5–39.5; 7.7–30.0). IVC diameters appeared to return to pre-donation values within 20 min but this was only detected by the hepatic long axis view.

**Conclusions:**

IVC diameter and collapsibility index variations, as measured in M mode, consistently detect volume changes after blood donation. The longitudinal mid-hepatic approach performed better by allowing a panoramic view, avoiding anatomical aberrancies at fixed points and permitting to identify the best possible perpendicular plane to the IVC. In addition, it was able to detect time-dependent physiological volume replacement. In contrast, in our hands, the renal window could not be visualized consistently well.

## Background

Changes of volume status can be readily inferred from variations in diameter of the inferior vena cava (IVC) with a bedside, non-invasive, repeatable point of care ultrasound scan, especially in the case of hypovolemia resulting from hemorrhage or other causes of acute intravascular depletion [1–3]. However, the effect size and time-course of IVC changes following acute blood loss are not fully established.

A widely used experimental model to reproduce haemorrhagic conditions in humans is blood letting in healthy donors but the absence of standard anatomical locations and scanning approaches pose a problem in assessing the reproducibility of IVC diameter measurements. Conflicting results were reported by Lyon et al. [4], Resnick et al. [5] and Juhl-Olsen et al. [6], who applied different scanning modalities and reference locations to the blood donor model. In this study, three different approaches to measuring IVC variables were compared in order to establish their relative ability to detect volume depletion after blood donation. The objective was to establish the most appropriate and reliable technique to identify volume-dependant changes in IVC diameters in healthy blood donors.

## Methods

### Study design

A convenience sample of 54 volunteer blood donors was enrolled after signing their informed consent. The study was approved by the hospital Ethics Committee and performed in accordance with the principles of the Helsinki declaration. Standard protocols for blood donation were followed. Inclusion criteria were: age 18–60, body weight >50 kg, hemoglobin >12.5 g/dl, body temperature <37 °C. Exclusion criteria were interruption of blood donation and onset of symptomatic hypotension.

### Study setting and population

The study was performed in the blood donation center of a metropolitan teaching hospital. All measurements were obtained in a reserved room next to the blood bank open space in order to assure privacy and minimize environmental stress. BMI was calculated as kg/m^2^. Only one volunteer had hypertension, treated by calcium channel blockers without diuretics.

The volunteers were supine during all pre- and post-donation ultrasound examinations. Measurements were obtained in M mode immediately before and as soon as possible after donation. The time elapsing from needle removal to beginning of the post-donation ultrasound scan, which depended on nurse protocols and local circumstances, was recorded.

**Fig. 1:**
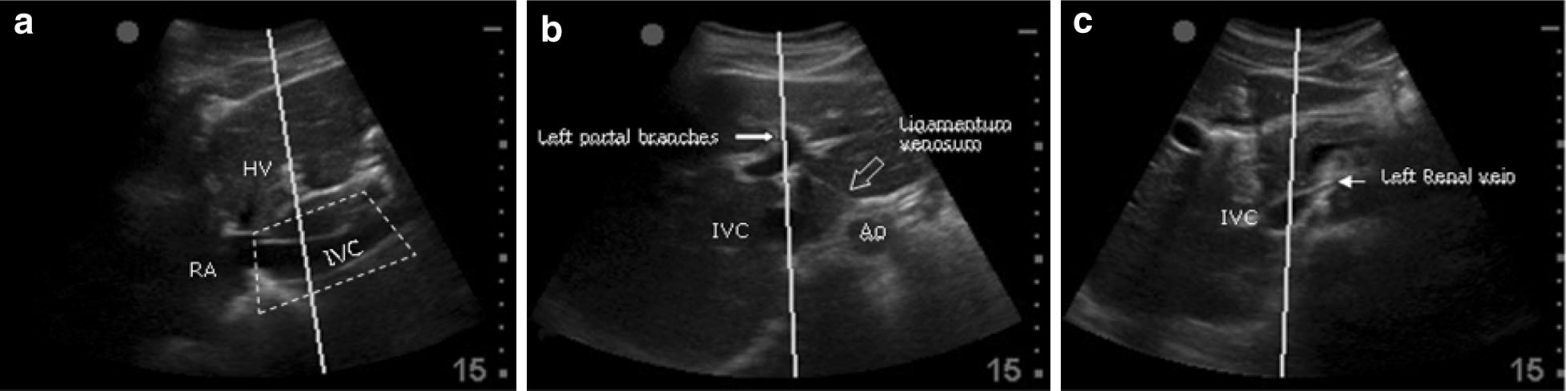
Mid-hepatic long axis (**a**), mid-hepatic short axis (**b**) and renal short axis (**c**) windows with M mode line for inferior vena cava (IVC) measurements **a** IVC diameter was calculated searching the best perpendicular approach, moving in a region (*dotted box*) below the hepatic vein (*HV*). *RA* right atrium. **b** *IVC* cross-sectional diameters which included the visualization of the ligamentum venosum and/or left portal branches as references points. *Ao* abdominal aorta. **c** *IVC* cross-sectional diameter at the left renal vein inlet

Inspiratory and expiratory IVC diameters were measured by an experienced sonographer (PP) in hepatic long axis, hepatic short axis and renal short axis views using a 2–5 MHz curvilinear probe (Sonosite M-Turbo; Sonosite Inc., Bothell, WA, USA). For the first two windows, the antero-posterior IVC diameters were calculated in both short and long axis by placing the probe in the subxiphoid region. In order to obtain the best possible right-angled approach to the longitudinal measure of IVC, the probe was moved downwards from the hepatic vein junction until the point with the best perpendicular view was located. The hepatic short axis was defined as the best round cross-sectional view of the IVC which included the left portal branches and/or the *ligamentum venosum* in the middle of the screen. The renal short axis was visualized at the level of the junction of the IVC with the left renal vein (Fig. 1). At least three measurements were taken for each mid-hepatic window and at least two for the renal window.

IVC measurements were first calculated in real time using the ultrasound software and subsequently on stored digital images in a post-processing study performed by a second operator (SA). Full recordings of all examinations were obtained by connecting the ultrasound machine to a portable personal computer using an open source software (Image J Open Source Software, National Institutes of Health, USA) [7]. Both real-time and post-processing measurement data were saved.

### Calculations

The maximum and minimum IVC diameters were calculated by tracking the distance between anterior and posterior walls in M mode. The IVC collapsibility index was calculated using the formula

$$\left[ ( {{\text{maximum}}\,\,{\text{IVC}}\,\,{\text{diameter}} - {\text{minimum}}\,\,{\text{IVC}}\,\,{\text{diameter}}} ) / {\text{maximum}}\,{\text{IVC}}\,{\text{diameter}} \right]$$

during a spontaneous breathing cycle. The real-time M mode IVC collapsibility index calculation was obtained on frozen screen images. The post-processing IVC diameters were taken after a frame by frame image selection, comparing the measuring scales of the ultrasound machine and Image J software.

### Statistical analysis

Each donor served as his/her own control. The study was designed to have an 80 % power to detect a 20 % difference in percentage collapse of the IVC between measurement locations with a level of significance of 0.05. Although the estimated sample size was 34, we planned to enroll 54 subjects to allow for dropouts due to possible difficulties in measurements at some particular windows and/or after blood donation. The agreement between methods was assessed by calculating the paired difference between them for each measurement and by estimating the bias (difference) and 95 % limits of agreements (LoA) relative to the mean measurement of both methods [8]. Non-constant differences between methods were detected by regressing differences on averages and checking whether the slope was 0. Non-constant variance was checked by regressing the absolute residuals on the averages, and checking whether the slope was 0 [9].

Changes in IVC diameters and collapsibility index after donation were analyzed using the paired *t* test. Normality of distribution was tested with the Wilk–Shapiro test. Mean values before and after donation was reported along with 95 % confidence intervals computed using bootstrap resampling (1000 repetitions). Associations between continuous variables were analyzed by Pearson’s correlation coefficient. Statistical significance was considered at *p* < 0.05.

Analyses were performed using the Statistical package R 2.15 (Foundation for Statistical Computing, Wien, Austria, 2012).

## Results

Out of 54 consecutive subjects, three were excluded, one for anemia, one for hypotension requiring rehydration after donation, and one for abnormal IVC dilatation/saber profile. Because of bowel gases, adequate IVC visualization was only achieved in 47 out of the other 51 donors with failure rates of 7.9, 11.8 and 41.2 % for the hepatic long axis, hepatic short axis and renal vein approach, respectively. Of the 47 subjects who were fully assessed 27 were males and 20 females, with a BMI of 24.42 ± 3.03. Out of these, 21 (45 %) donated 400 ml blood bags, 1 (2 %) a 410 ml bag, and 25 (53 %) 450 ml blood bags. The weighted average blood donation for the entire sample was 426 ml.

**Fig. 2:**
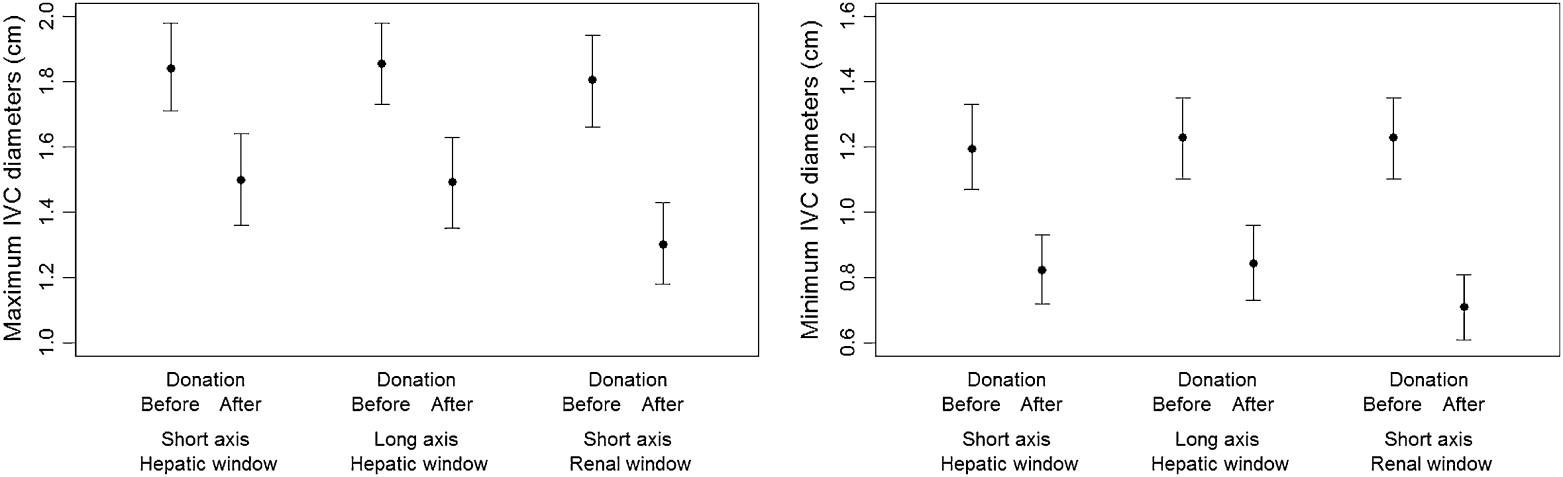
Maximum (*left*) and minimum (*right*) pre- and post-donation expiratory and inspiratory absolute diameters with three different windows

The 95 % limits of agreement between real-time and post-processing measurements are reported in Table 1. No significant differences were found between real-time and post-processing measurements at the hepatic and renal windows. The mean maximum/minimum pre-donation IVC diameters were 1.86/1.22 cm for the middle hepatic long axis, 1.83/1.17 cm for the middle hepatic short axis, and 1.81/1.15 cm for the renal short axis. All mean IVC diameters decreased [−19.9, −18.0, −26.5 %; absolute change: 0.37 cm; 0.33 cm; 0.48 cm in maximum diameters; −31.1, −1.6, −36.5 %; absolute change: 0.38; 0.37; 0.42 cm in minimum diameters] after donation (Fig. 2; Table 2).

**Table 1:** Average bias (average of the differences between values measured in real time and values measured in post-processing)

	Mean (real time measurement)	Mean (post-processing)	Average bias	95 % limit of agreement	*p* value for trend	*p* value for variability consistence
*Short axis hepatic window*						
Max diameter before donation	1.77	1.99	–0.04	−0.41; 0.33	0.34	0.27
Min diameter before donation	1.15	1.17	0.01	–0.32; 0.35	0.19	0.11
Max diameter after donation	1.54	1.71	0.09	−0.25; 0.42	0.98	0.5
Min diameter after donation	0.74	0.72	0.09	−0.22; 0.39	0.7	0.81
*Long axis hepatic window*						
Max diameter before donation	1.88	1.95	–0.06	−0.47; 0.35)	0.48	0.06
Min diameter before donation	1.26	1.26	0.02	−0.34; 0.38	0.37	0.62
Max diameter after donation	1.56	1.56	0.04	−0.23; 0.32	0.59	0.07
Min diameter after donation	0.83	0.82	0	Not constant	0.99	0.03
*Short axis renal window*						
Max diameter before donation	1.65	1.82	0.02	−0.36; 0.40	0.51	0.51
Min diameter before donation	1.15	1.16	Not constant	−0.39; 0.46	0.01	0.76
Max diameter after donation	1.34	1.3	0.02	−0.7; 0.74	0.27	0.67
Min diameter after donation	0.66	0.59	0.06	Not constant	0.34	0.04

Trend was assessed by regressing differences (between methods) on averages and checking with a *t* test whether the slope was 0; variability consistence was assessed by regressing averages (between methods) against the absolute residuals computed from the regression model used for trend and checking with a *t* test whether the slope was 0

**Table 2:** Results with three windows scan

Study variables	*N*	Pre		Post		Absolute change		Percent change		*p* value*
		Mean	95 % CI	Mean	95 % CI	Mean	95 % CI	Mean (%)	95 % CI	
*Long axis hepatic window*										
IVC la max (cm)	47	1.86	1.73–1.99	1.49	1.35; 1.63	−0.37	−0.27; −0.45	−19.9	−24.1; −14.5	<0.0001
IVC la min (cm)	47	1.22	1.09–1.34	0.84	0.72; 0.97	−0.38	−0.26; −0.49	−31.1	−40.1; −21.3	<0.0001
IVC la CI (%)	47	35.2	31.2–39.2	44.9	40.0; 49.8	9.7	4.1; 15.1	21.6	11.6; 42.9	<0.001
*Short axis hepatic window*										
IVC sa max (cm)	45	1.83	1.70; 1.96	1.50	1.35; 1.64	−0.33	−0.24; −0.42	−18.0	−22.9; 13.1	<0.0001
IVC sa min (cm)	45	1.17	1.05; 1.29	0.80	0.69; 0.91	−0.37	−0.22; −0.53	−31.6	−45.2; −18.8	<0.0001
IVC sa CI (%)	45	36.2	32; 40.4	46.8	43.0; 50.5	10.6	6.7; 14.3	22.6	18.5; 39.5	<0.0001
*Short axis renal window*										
IVC Ren max (cm)	30	1.81	1.65; 1.96	1.33	1.17; 1.49	−0.48	−0.29; −0.65	−26.5	−35.9; −16	<0.0001
IVC Ren min (cm)	30	1.15	1.00; 1.30	0.73	0.62; 0.83	−0.42	−0.27; −0.53	−36.5	−46.0; −23.4	<0.0001
IVC Ren CI (%)	30	37.7	31.5; 43.8	46.7	41.4; 51.9	9.0	2.9; 15.1	19.3	7.7; 30.0	<0.006

*IVC* inferior vena cava, *LA* long axis, *SA* short axis, *CI* collapsibility index

* Paired *t* test

**Fig. 3:**
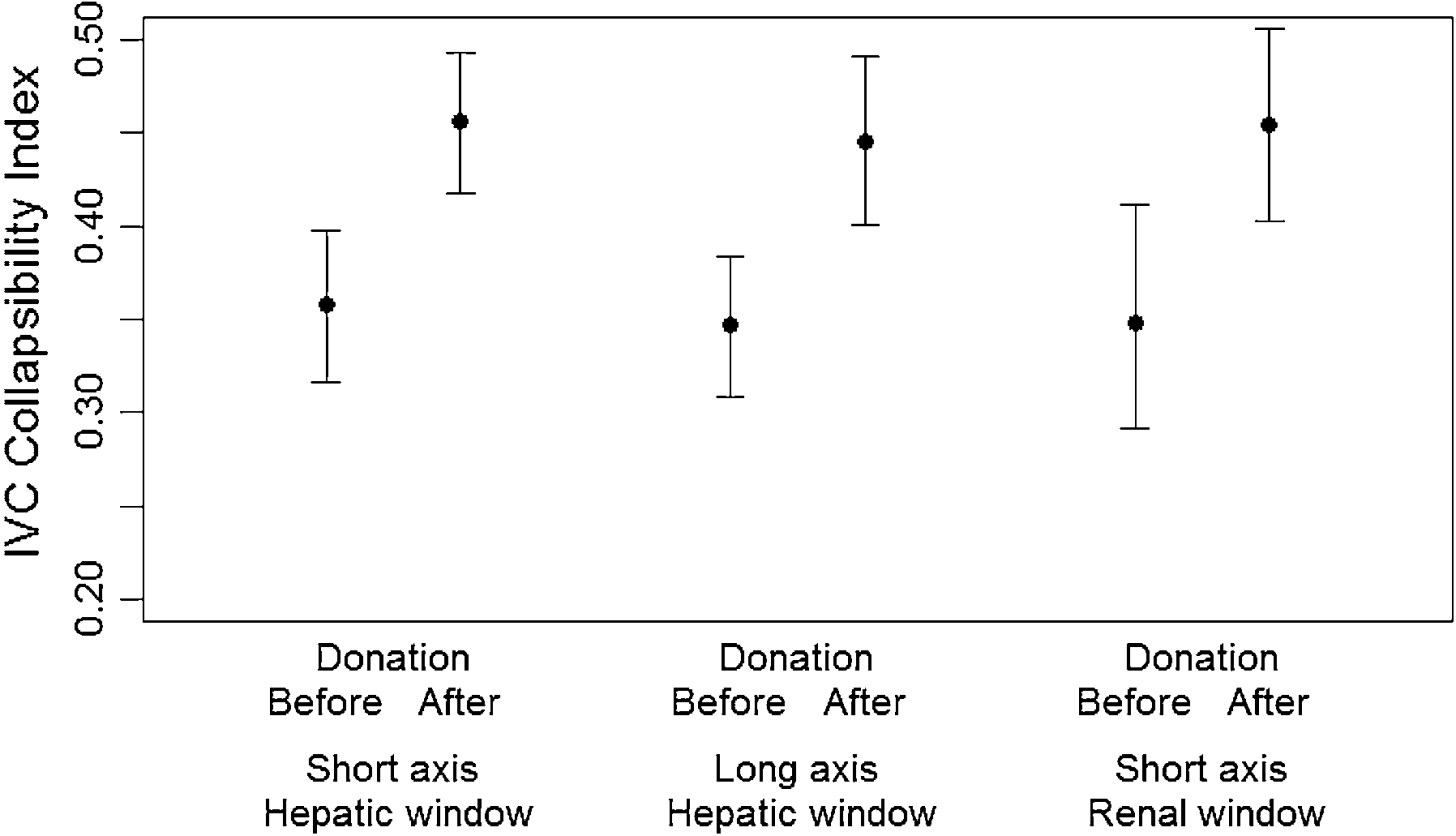
Before and after donation IVC collapsibility index values measured by three different approaches

The mean pre-donation collapsibility indexes were 0.35 [0.31–0.39], 0.36 [0.32–0.40] and 0.37 [0.31–0.43] for the middle hepatic long axis, middle hepatic short axis, and renal short axis, respectively. The mean collapsibility index increased (+9.7, +10.6, +9.0; 95 % CI: 4.1–15, 1; 6.7–14.3; 2.9–15.1; absolute change: +21.6, +22.6, +19.6 %) for each window after blood donation (*p* < 0.001 for the two hepatic windows, and *p* < 0.006 for the renal window) (Fig. 3; Table 2).

**Fig. 4:**
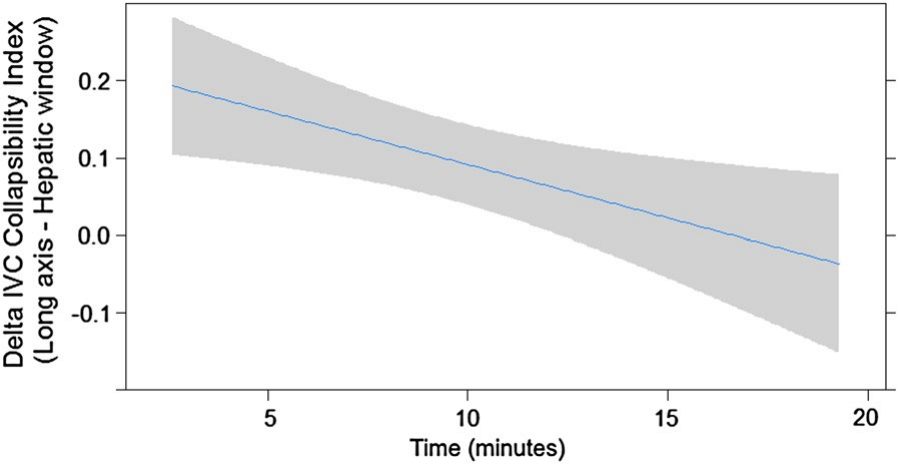
Time course of Delta-IVC collapsibility index, as measured in hepatic window—long axis, showing return to baseline values within 20 min after end of blood donation

The mid-hepatic long axis IVC collapsibility index revealed a time-dependent decrease after donation, maximum values being measured soon after blood letting and gradually decreasing to no difference from pre-donation levels within 20 min of removing the needle (Fig. 4).

## Discussion

This study reveals that an experienced operator was able to detect consistent changes in IVC absolute diameters and collapsibility index in healthy donors after an average 426 ml blood loss, by performing serial ultrasound measurements. Confirmatory, blinded measurements in post-processing allowed another operator to validate the data collected in real time.

Little information is available on the complex pathophysiology of volume regulation after blood donation. Rapidly increasing sympathetic activity and decreasing vagal activity were reported in a post-donation setting [10] and slower refill from the interstitial space of up to one liter per hour after bleeding was reported, although in military settings [11]. These phenomena may reflect on IVC diameters and collapsibility index, but there is little agreement among previous studies (4–6), possibly due to methodological factors, which we addressed in this paper by using three different windows to identify variations of blood volume. In our hands the renal window was assessable in only 58.8 % of subjects, due to bowel gases, whereas the hepatic long axis window had the highest visualization rate and was the only one able to detect a time-dependent post-donation physiological volume replacement.

Different methodologies are reported in the literature to measure IVC diameters, depending on probe selection (convex or sector), orientation (longitudinal or transversal) and measurement site along the vessel (proximal, mean or distal sub-xyphoid scan) [6, 12–16]. Another variable is represented by only measuring IVC maximum and minimum diameters, rather than calculating the collapsibility index as in this paper. In addition, anatomical variations of IVC diameters, such as sub-diaphragmatic abnormal enlargement (saber profile) [17], or fixed points taken at the diaphragm and hepatic vein crossing can interfere with vein compliance and studies of IVC collapsibility [16]. Finally, a recent report describing up to 21 mm cranio-caudal longitudinal IVC displacements associated with the respirophasic cycle highlights additional potential errors in computskippinging maximum and minimum diameters [18].

For these reasons, we compared multiple measurements of the IVC at different levels using different windows. In addition, a larger sample than reported in previous studies in blood donors was investigated. We used a convex probe to achieve a sequence of three IVC points of view at mid-hepatic long axis, mid-hepatic short axis and renal short axis. Rather than considering a single reference point in the longitudinal scan (such as the right atrial-IVC junction, 2 cm under the right atrium, hepatic vein junction, or 2 cm below the hepatic vein junction) [4–6, 12, 13, 19–21] we moved along the IVC below the hepatic vein junction, trying to achieve the best possible perpendicular approach.

Measurements were performed both in real time, as one would in a clinical setting, and post-processing on recordings obtained by dedicated software [7], in order to validate and increase the number of measurements. The concordance between real-time and post-processing imaging (Table 1) was satisfactory, thus validating measurements collected in the former condition.

Changes in IVC diameters were observed after blood donation (Table 2; Fig. 2), similarly to the reports by Lyon et al. [4], Resnick et al. [5], and Juhl-Olsen et al. [6]. In our study, as IVC diameters decreased, a significant increase in collapsibility indexes was detected by all IVC approaches (Fig. 3; Table 2), similarly to Juhl-Olsen et al. [6] and differently from Resnick et al. [5]. As suggested above, probe type (sector), site (right atrial IVC or 2 cm below hepatic inlet) and a single calculation, as used by some authors [4, 6], could account for the different results.

Finally, in our study the mean baseline collapsibility index values before blood donation, 35.2 % (95 % CI: 31.2–39.2) in hepatic long axis and 36.2 % (95 % CI: 32–40.4) in hepatic short axis, were similar to those reported by Minutiello [21] and Weekes et al. [22], suggesting that these values may represent the normal range in healthy subjects. Likewise, a similar IVC collapsibility index, 0.37 (95 % CI: 0.31–0.43), was measured with the renal approach, suggesting homogeneous oscillations of a wide region of the vessel, in accordance with Wallace et al. [16]. Those authors found equivalent rates of IVC collapse at the levels of the left renal vein and 2 cm caudal of the hepatic vein inlet (35 % vs 30 %), whereas measurements taken at the right atrial junction, resulted in a lower collapsibility index, 20 %, possibly due to higher rigidity of the IVC in the vicinity of the diaphragm [16]. Consequently, we assumed that M mode mid-hepatic measurements taken some centimeters from the fixed point represented by the hepatic vein inlet would be more uniform, in spite of IVC displacement.

Another objective of this study was to identify which window performs better in detecting moderate blood loss. The hepatic window (long and short axis) permitted better visualization of IVC diameters than the renal one, which could be correctly identified in only 30 out of 47 subjects. The hepatic window in short axis was visualized in 45 patients, whereas the hepatic window in long axis performed well in 47. Furthermore, measurements of the collapsibility index in mid-hepatic long axis correlated significantly with the time elapsed from the end of blood donation (Fig. 4), suggesting that this is the best approach to provide data associated with the physiology of volume recovery, as described in studies performed using post-donation haematocrit analysis [23]. Subjects examined with this window had a mean rise in IVC collapsibility of about 20 % soon after the end of blood donation, which progressively returned to baseline values within 20 min (Table 2).

Strengths of this study include the comparative use of three different windows to evaluate changes in IVC diameters, the observation of a sample larger than in previous studies and the recording of time from end of donation to post-bleeding assessment. In addition, normal BMI in blood donors facilitated IVC diameters acquisition. Potential limitations are that while the subjects studied were healthy voluntaries with expected normal central venous pressure (CVP), patients with chronically elevated CVP may respond differently to acute volume depletion. In addition, IVC diameters were measured in a quiet experimental environment, not necessarily resembling that of an Emergency Department. Monitoring patients by ultrasound includes serial measurements of the IVC. In this respect, in our hands, real-time measurements of IVC diameters and collapsibility index proved as reliable as those obtained when processing serial recordings, suggesting that they may be applied even within the time constraints of an emergency situation. In addition, a simple, inexpensive and reliable software could be of advantage, as images can be easily transferred to a standard portable computer and examined by other operators to improve diagnostic reliability. Finally, we performed measurements in M mode only and did not compare them with the B mode in the same window.

## Conclusions

This study suggests that volume depletion after blood donation can be detected by measuring IVC diameters and collapsibility index variations in M mode. There were no differences between IVC longitudinal and cross-sectional mid-hepatic windows. However, the longitudinal mid-hepatic scans allowed to obtain a panoramic view of the IVC under the hepatic vein inlet, to avoid anatomical aberrancies at fixed points, and to identify the best possible perpendicular approach in a relatively long segment of the vessel, thus reducing the risk of errors or paradoxical effects. In contrast, assessing one single point along the vessel and taking one single measurement of the IVC could lead to unreliable results. Finally, in our hands, the longitudinal mid-hepatic IVC collapsibility index detected a phenomenon consistent with time-dependent physiological volume replacement. We suggest that visualizing the hepatic long axis minimizes errors by selecting and taking a perpendicular approach to the best point of interest. Further studies are needed to improve standardization of the site of IVC measurements and to validate the role that IVC assessment plays in the early ultrasound diagnosis of blood loss to avoid systematic bias and consequent errors in clinical and especially critical settings.

## Abbreviations

IVC: inferior vena cava
BMI: body mass index
CVP: central venous pressure
